# Using TikTok for public and youth mental health – A systematic review and content analysis

**DOI:** 10.1177/13591045221106608

**Published:** 2022-06-10

**Authors:** Darragh McCashin, Colette M Murphy

**Affiliations:** 8818 Dublin City University, Dublin, Ireland

**Keywords:** Review, tiktok, psychology, qualitative, children

## Abstract

Globally, TikTok is now the fastest growing social media platform among children and young people; but it remains surprisingly under-researched in psychology and psychiatry. This is despite the fact that social media platforms have been subject to intense academic and societal scrutiny regarding their potentially adverse effects on youth mental health and wellbeing, notwithstanding the inconsistent findings across the literature. In this two part study, we conducted a systematic review concerning studies that have examined TikTok for any public health or mental health purpose; and a follow-up content analysis of TikTok within an Irish context. For study 1, a predetermined search strategy covering representative public and mental health terminology was applied to six databases – PSYCINFO, Google Scholar, PUBMED, Wiley, Journal of Medical Internet Research, ACM – within the period 2016 to 2021. Included studies were limited to English-speaking publications of any design where TikTok was the primary focus of the study. The quality appraisal tool by Dunne et al., (2018) was applied to all included studies. For study 2, we replicated our search strategy from study 1, and converted this terminology to TikTok hashtags to search within TikTok in combination with Irish-specific hashtags. As quantified by the app, the top two “most liked” videos were selected for inclusion across the following three targeted groups: official public health accounts; registered Irish charities; and personal TikTok creators. A full descriptive analysis was applied in both studies. Study 1 found 24 studies that covered a range of public and mental health issues: COVID-19 (*n* = 10), dermatology (*n* = 7), eating disorders (*n* = 1), cancer (*n* = 1), tics (*n* = 1), radiology (*n* = 1), sexual health (*n* = 1), DNA (*n* = 1), and public health promotion (*n* = 1). Studies were predominately from the USA, applied a content analysis design, and were of acceptable quality overall. In study 2, 29 Irish TikTok accounts were analysed, including the accounts of public health authorities (*n* = 2), charity or non-profit (*n* = 5), and personal TikTok creators (*n* = 22). The overall engagement data from these accounts represented a significant outreach to younger populations: total likes *n* = 2,588,181; total comments *n* = 13,775; and total shares *n* = 21,254. TikTok has been utilised for a range of public health purposes, but remains poorly engaged by institutional accounts. The various mechanisms for connecting with younger audiences presents a unique opportunity for youth mental health practitioners to consider, yet there were distinct differences in how TikTok accounts used platform features to interact. Overall, there is an absence of high quality mixed methodological evaluations of TikTok content for public and mental health, despite it being the most used platform for children and young people.

## Introduction

The pervasive use of social media worldwide has generated an ideal platform for promoting public and mental health information to children and young people (CYP). Amidst the ongoing global pandemic, coupled with evidence of increasing youth psychological difficulties, public health officials and clinicians are increasingly recognising the need to understand and engage social media including TikTok for public communications (Eghtesadi & Florea, 2020).

Recent reviews have found that popular social networking sites are the most important digital health resources for CYP (Lupton, 2021; Montag et al., 2021). Where supply exceeds demand for in-person mental health services, digital solutions can provide stakeholders with the opportunity to engage with large portions of the youth population with informative content (Liu et al., 2020). However, there is a limited understanding of the functionality of TikTok among mental health professionals. Based on receptiveness to other dominant social media platforms, this lack of understanding may be attributable to: the speed of the cultural embeddedness of TikTok; generational differences in uptake, digital literacy and competencies; and a traditional scepticism of new technologies amongst professionals.

### What is TikTok?

TikTok allows users to consume and create short videos between 15–60 s in length; using various filters, music and lip-syncing templates. TikTok’s unique selling point is that the content presented to an individual is algorithm-driven, and tailored to their indicated preferences and previously liked content (Anderson, 2020). It is particularly popular with the traditionally hard-to-reach 13–29 age cohort – data from the United States show that 32.5% of users are aged 10–19, and 29.5% aged 20–29 (Clement, 2020). Globally, it is assumed that the majority of TikTok users are of pre-teen age.

TikTok has been conceptualised as a highly unique video-based social media app with distinct technical structures and unparalleled user adoption unlike any other platform, thereby making it a specific online network wherein imitation and memetic features further accelerate its diverse user interactivity (Zulli & Zulli, 2020). As a result, there have been waves of viral TikTok videos among CYP, with accompanying hashtags relating to topical issues, including COVID-19, mental health, eating disorders, developmental issues, and health (Montag et al., 2021). Indeed, despite the notable lack of psychology professionals effectively utilising TikTok, there have been interesting exceptions. For example, Dr. Julie Smith is considered to be one of the first accredited psychologists to successfully use TikTok to disseminate mental health information on a range of topics from anxiety to suicidality. At present, her TikTok profile has garnered 33.4 million likes and amassed over 2.9 million followers, from presenting bite-sized psychoeducational content from her professional background (Smith, 2022).

### Critically appraising the role of TikTok

Across the social media landscape, there has been heated debate about the potentially harmful effects of popular platforms, including Facebook, Instagram and Snapchat. More broadly, the overall effect of screentime itself on CYP has been under extensive investigation. Several high profile media exposes have prompted clinicians and parents to re-evaluate the relationship between social media and the CYP. For example, a recent article in The Wall Street Journal leaked internal research by Facebook (who also own Instagram) purporting to show that the platform holds evidence demonstrating the harms caused to the mental health of approximately 20% of its users. However, upon closer inspection, the referenced research reflects much of the characteristics in the broader literature on the clinical effects of social media consumption on CYP (insert Ritchie). That is, the overall studies are largely correlational, overly reliant on self-reported and thus unreliable measures, lacking in construct validity, and of significantly varying quality to draw any definitive conclusions.

Although there is no specific clinical literature on TikTok use, similar concerns exist given its significant pre-teen userbase (Bucknell Bossen & Kottasz, 2020). Expectedly, the same challenges regarding the online safety, content moderation, data regulation and the ethics of targeted advertising of CYP are applicable to TikTok (De Leyn et al., 2021). For example, a recent content analysis found that alcohol-related content on TikTok showed a propensity to promote rapid consumption of multiple drinks and to align alcohol consumption with positive associations (i.e., humour), while rarely communicating the known negative outcomes (Russell et al., 2021).

From a theoretical standpoint, the unique mimicry and imitation in-built into TikTok creates a new form of interaction between users and video content – the perceived realism of which could lead to behavioural change in CYP, in line with the principles of social learning theory (insert REF). Of the scant research available, data suggests that user motivations – archiving, self-expression, social interaction and peeking – were significant predictors of TikTok behaviours more so than personality traits (Omar & Dequan, 2020). Relatedly, a frequently applied theoretical framework to TikTok research is that of the uses and gratifications approach (Montag et al., 2021) which states that individuals use media in particular ways to satisfy their own needs, and thus feel satisfied and engaged (Katz et al., 1974). In applying this theory, recent survey research from China (*n* = 1051) found that novelty was the distinct gratification point for TikTok use across all users (Scherr & Wang, 2021) – that is, TikTok’s algorithm continually produced novel yet relevant content outside of the user’s immediate social network.

As a platform, TikTok has acknowledged the positive and negative uses of their technology; and has recently issued advice about overusing the app at night, in addition to providing resources to users searching for suicide-related content (TikTok, 2020, 2021).

### The present study

To address the gap in the psychology and psychiatry literature on TikTok, this study set the following two aims:

1) Conduct a systematic review on the use of TikTok for any public and mental health purpose
2) Contextualise and supplement the review findings with a content analysis, using an Irish specific case study.

## Method

### Study 1

#### Search strategy

**Figure 1.**
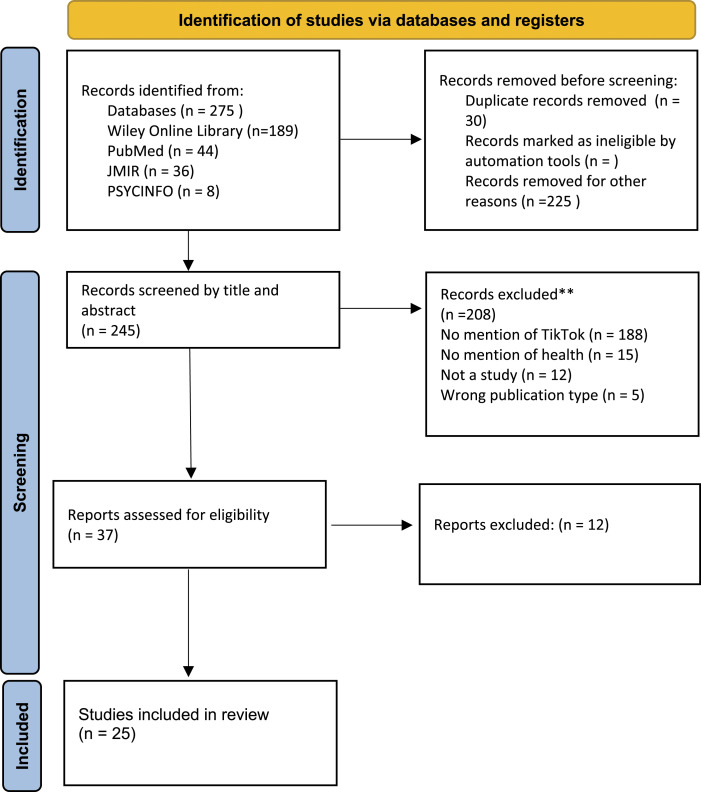
PRISMA flow chart for study 1.

A predetermined search strategy covering representative public and mental health terminology and blended with the word TikTok was applied to six databases – PSYCINFO, PUBMED, Wiley, Journal of Medical Internet Research (JMIR),– within the period 2016 to 2021. This is fully detailed in our supplementary materials. The search was conducted between June and July 2021, with 275 studies initially screened. The flow diagram for the identification of studies is provided in our PRISMA in Figure 1 (Page et al., 2021).

#### Selection criteria and quality appraisal

Included studies were limited to English-speaking publications of any design where TikTok was the primary focus of the study. The quality appraisal tool by Dunne et al. was applied to all quantitative studies (Dunne et al., 2018); and the CASP (2018) checklist was applied to all qualitative studies. The second author (CM) conducted full title and abstract screening, with both authors then deliberating until consensus regarding any articles with unclear eligibility.

### Study 2

#### Search strategy and data collection

We sought to replicate our search strategy from study 1, and thus converted this terminology to TikTok hashtags to search within TikTok in combination with Irish-specific hashtags. As quantified by the app, the top two “most liked” videos were selected for inclusion across the following three targeted groups: official public health accounts; registered Irish charities; and personal TikTok creators. This was conducted on a self-made research-only TikTok account during July 2021. The full strategy is provided in our supplementary materials. Table 1 provides a summary of the overall TikTok accounts and features from this search strategy.

**Table 1.** Summary table of overall TikTok features from the search strategy.

Video categories - How many videos and video source	Video	Type of public health content	Verified accounts	Followers
Category 1: This was HSElive		Covid (n = 2)	Verified - 2	
n = 2 - public health official	1. @hselive	Covid	Y	17.3k
	2. @hselive	Covid	Y	23.8k
Category 2: A range of established registered charities across Ireland were included		Covid (n = 1)	Verified - 1	
		Cancer (n = 1)		
		Mental Health (n = 3)		
N=6 - charities	3. Spunout	Covid	Y	1990
	4. Irishcancancersociety	Cancer	N	25
	5. @Jigsaw_ymh	Mental Health	N	79
	6. @Belongtoyouthservice	Mental Health	N	5376
	7. @turn2me	Mental Health	N	35
Category 3: This range of TikTok videos was found from searching relevant hashtags on TikTok		-Physical topics: Asthma; Skin Cancer; Diabetes; Oral Health; Allergies (n = 6)	Verified - 0	
		-Covid (n = 1)		
		-Car Crash (n = 1)		
		-First Aid (n = 1)		
		-Substance Use: smoking; alcohol (n = 2)		
		-Mental Health: General; Body Image; Bipolar; Depression; Anxiety; Eating Disorder (n = 8)		
		-Sexual Health (n = 1)		
		-Fitness: Nutrition; Fitness (n =2)		
N = 21 - personal accounts	8. #irish #carcrash	Car Crash	N	795
	9. #irish #firstaid @classycody	First Aid	N	237
	10. #irish #smoking @lilgeep	Smoking	N	5020
	11. #irish #alcohol ‘shanejkennedy	Alcohol	N	199.2k
	12. #irish#mentalillness @saoishell	Mental Health	N	150.2k
	13. #irish #mentalhealth @benzoooz	Mental Health	N	574.3k
	14. #irish #depressed @arachnerd_cosplay	Mental Health	N	930
	15. #irish #anxiety @wonder_art	Mental Health	N	17.9k
	16. #irish #edawareness @eimearmc9	Mental Health	N	5607
	17. #irish #bipolar @cymbaltaslut	Mental Health	N	204
	18. #irish #bodyimage @oxrobyn.sucksxo	Mental Health	N	1193
	19. #irish #sexual health @accidentalainbow	Sexual Health	N	4976
	20. #irish #vaccine @killersundy	Covid-19	N	58.1k
	21. #irish ∼dentist	Oral Health	N	3654
	22. #irish #asthma	Asthma	N	4411
	23. #irish #allergy @irishmammylife	Allergy	N	5839
	24. #irish #skin @.sophiekeegan	Skin	N	94.4k
	25. #irish #cancer @bernadettehagans	Cancer	N	111.3k
	26. #irish #diabetes @hi_im_lee	Diabetes	N	1534
	27. #irish #nutrition @@michneilly	Nutrition	N	189.0k
	28. #irish #health @tadghoody	Fitness	N	1129.1k

#### Coding approach

A full descriptive table was built a priori to centralise all useful information across our findings. This included engagement data, such as number of likes, followers, comments; in addition to video characteristics, summaries, and length. Similar to other comparable studies, the presence of dialogic loop was deemed important and relevant to TikTok in particular – this refers to the posing and answering of questions to promote further engagement (Chen et al., 2021; Wang & Yang, 2020). As such, a frequency count was performed across all included content. The second author manually performed all data extraction and synthesis using Microsoft Excel.

## Results

### Study 1

A total of 24 studies were included in the final review. These studies were predominantly from the USA (*n* = 20), with the remaining studies from China, Ireland, Australia, and Canada. The majority of studies adopted a form of content analysis as their primary research methodology (*n* = 20), with other studies utilising cross-sectional design (*n* = 1), thematic analysis (*n* = 2), or cases series (*n* = 1). A broad range of topics were found across the TikTok research landscape, including COVID-19 (*n* = 10), dermatology (*n* = 7), eating disorders (*n* = 1), cancer (*n* = 1), tics (*n* = 1), radiology (*n* = 1), sexual health (*n* = 1), DNA (*n* = 1), and public health promotion (*n* = 1). The overall quality of studies was variable – with the majority of studies being of either low (*n* = 11) or acceptable (*n* = 7) quality, leaving 6 studies appraised as good quality. A full breakdown of all study characteristics and quality appraisal outcome is provided in Table 2.

**Table 2.** Table of study characteristics (Study 1).

Authors (year)	Country	Main Objectives	Population characteristics	Study Design	Key Results	Category	Quality of Study
Basch et al. (2020)	USA	To describe the content of COVID-19 TikToks.	Covid TikToks	content analysis	Anxiety and quarantine were the most common topics covered. Fewer than 10% of videos mentioned virus transmission, symptoms, or prevention.	Covid-19	Acceptable
Basch et al. (2021)	USA	To describe COVID-19 vaccination content on TikTok	Covid vaccination videos	Content analysis	Videos that encouraged a vaccine got over 50% of total cumulative views and just under 50% of the total likes. The majority of videos discouraging a vaccine showed a parody of an adverse reaction.	Covid-19	Good
					Twenty-two of these 38 videos (57.89%) falsely conveyed that a vaccine was available, as they were not at the time of the study.		
Basch, Fera, & Quinones (2021)	USA	To describe the content of DTC DNA testing videos on TikTok.	DTC DNA TikTok	Content Analysis	Majority mentioned finding family roots (94%), commercial DNA testing kits (67%), and fa person taking/talking about taking a DNA test (92%).	DNA	Acceptable
					App features: Neither the use of music nor the mention of using DNA testing to locate family had a significant effect on a video's comments or likes.		
Basch, Fera, Pierce, et al. (2021)	USA	Examine the characteristics of TikTok videos related to mask wearing.	Mask TikToks	Content Analysis	Videos with hashtag #WearAMask generated 500 million views, videos posted by WHO generated 57 million views. #WearAMask videos received Almost 10 times as many cumulative views as WHO videos.	Covid-19	Acceptable
					App features: 68% of #WearAMask videos involved humour. 9% of the WHO videos involved humour. 27% of #WearAMask videos involved dance whereas none of the WHO videos involved dance.		
Basch, Mohlman, Fera, et al. (2021)	USA	To describe TikToks related to COVID-19 testing.	Covid-testing TikToks	Content analysis	Even though only 44/100 videos portrayed tests as disgusting/unpleasant, they gathered over 70% of the total cumulative number of views and likes.	Covid-19	Good
					Those that mentioned or suggested anxiety attracted about 60% of the total cumulative number of views and more than 8 million likes.		
Baumel et al. (2022)	USA	To examine TikTok mask effectiveness misinformation.	Mask TikToks	Content Analysis	5% of videos contained health professionals, 17.67% contained misinformation, 4% cited scientific journals.	Covid-19	Low
					In the “WearAMask” hashtag 24% contained healthcare professionals, 10.6% contained misinformation and 5.3% cited scientific journals.		
					In the “MasksDontWork” hashtag, 1.33% contained healthcare workers, 45.3% contained misinformation and 2.67% cited scientific journals.		
Boatman et al. (2021)	USA	To determine characteristics that might contribute to a dermatology video's “virality”	Dermatology TikToks	Content analysis	Most TikToks were pro-vaccine, but anti-vaccine TikToks had more user interactions.	Sexual Health	Acceptable
					Pro-vaccine TikToks mainly covered cancer and prevention. Side effects were the main concern of antivaccine messages.		
					Approximately 30% of all top TikToks analysed were created by health professionals.		
Chen et al. (2021)	China	To determine the factors and influencing mechanisms related to citizen engagement with the TikTok account of the National Health Commission of China during the COVID-19 pandemic.	TikTok videos from National Health Commission of China	content analysis	The majority of videos related to guidance, information concerning the government's handling of the pandemic, the latest news about COVID-19, and appreciation toward frontline emergency services.	Health Promotion	Good
					Video length was negatively associated with citizen engagement. Title length was positively associated with citizen engagement. Dialogic loop was negatively associated with citizen engagement.		
					Emotional Valence: Longer videos with positive titles received more likes and comments. Videos related to the latest news received more likes if the video title displayed higher levels of positive emotion.		
Chen et al. (2021)	Australia	Finding out the misinformation around isotrestinoin on TikTok	Isotretinoin-related TikToks	cross-sectional study	Majority of videos did not include medical information. 48 (13.7%), 53 (15.1%) and 14 (4.0%) videos provided poor-quality, fair-quality and good-quality medical information respectively. Most of the videos categorised under “no medical information” (n = 211, 89.8%) were non-verbal montages of acne improvement.	Dermatology	Low
					Benefits of isotretinoin were covered in 18 videos (5.1%). Laboratory monitoring was mentioned in 12 videos (3.4%) and dosages were mentioned in 6 videos (1.7%).		
Galamgam & Jia (2021)	USA	To understand how teenagers and young adults use TikTok to engage with acne and isotretinoin information.	teenagers and young adults on TikTok	content analysis	Most videos focused on before and after improvement in acne severity while video comments primarily discussed side effects and personal anecdotes.	Dermatology	Low
Herrick et al. (2021)	Canada	Objective To explore eating disorder (ED) recovery-related content create and shared on the social media platform TikTok.	Creators on TikTok using #EDrecovery	thematic analysis	6.33% of videos were filmed by health care professionals, and 2.66% were filmed by young adult patients tracking their recovery journeys. 15.66% of videos communicated pragmatic health information and .66% provided misleading or inaccurate health advice, with the remaining videos depicting everyday quarantine activities in a satirical manner. Although videos by health care professionals were few in number, they were often among the most highly engaged with. Some of the posts walked the line between ED recovery and pro-ED content.	Eating Disorders	Good
Hull & Parnes (2021)	USA	To describe six teenage girls who developed functional tics after exposure to a single social media personality	Teenage girls on tiktok with tics	Case series based on medical records	Results suggest social media may contribute to the spread of functional neurologic symptom disorder, in a way previously requiring physical proximity	Tics	Acceptable
Kassamali et al. (2021)	USA	To determine what characteristics might contribute to a dermatology video's “virality	TikTok videos	content analysis	The content of the TikToks included treatment (71%,), patient empathy (52%,), diagnosis (26%), and disease etiology (23%)	Dermatology	Low
					Audiovisual features: most videos included on-screen text (90%), dermatologists wearing scrubs and/or white coats (68%) music (65%, 20/31), and procedural demonstrations (42)		
Li et al. (2021)	USA	To explore TikTok as a way to communicate COVID-19 information.	TikTok Videos	Content analysis	Subtitles were positively associated with the number of shares. A higher number of hashtags was associated with more likes. Dance videos had the most shares. Videos displaying alarm/concern, COVID-19 susceptibility/severity, precaution response efficacy had higher engagement rates.	Covid	Good
Lovett et al. (2021)	USA	To analyse the top radiology-related posts on TikTok in order to describe opportunities for radiology engagement.	Radiology-related TikTok videos	content analysis	Most posts (81%) were non-doctor radiology personnel, while only 5% were radiologists. Posts by radiologists had more plays than those by nonphysician radiology personnel. Posts by radiologists were mostly clinical (65%,). Posts about COVID-19 represented 38% of the study sample and 48% of posts after the first U.S. COVID-19 case.	Radiology	Good
Ostrovsky & Chen (2020)	USA	To analyse Covid-19 medical information communication across the three largest COVID-19–related categories on TikTok.	Covid-19 TikToks	content analysis	6.33% of videos were filmed by health care professionals and 2.66% by young adults communicating their recovery journeys. 15.66% of videos communicated pragmatic health information. 66% provided misleading or inaccurate health advice. Although videos by health care professionals were few in number, they were often among the most widely “liked” and shared across the board.	Covid	Low
Presley et al. (2022)	USA	To assess the audience engagement generated by top dermatologist influencers on Instagram Reels and TikTok.	Dermatologist infleuncers on TikTok	Content Analysis	Dermatologist influencer followers were 3,900,000 on TikTok. TikTok dermatologists get higher maximum post engagement (5.09%) in comparison to Instagram. TikTok videos were predominantly educational (20/30, approximately 67%).	Dermatology	Low
Presley et al. (2022)	USA	To determine study the top dermatologist influencers on TikTok.	Top 10 TikTok dermatologists	content analysis	The top 10 TikTok dermatologists include 3 dermatology residents, with 7 to 8 average years of practice among the remaining 7 influencers.	Dermatology	Low
					Each dermatologist's 3 most recent posts were exained:70% were educational, and educational posts had the highest mean user engagement. 23.33% of posts were “duets”, usually in response to highly-viewed videos created by other users. Duets were all educational and averaged 340,000 views		
Roche et al. (2021)	Ireland	To evaluate TikTok from a dermatology health-promotion perspective.	TikTop dermatology videos	Content analysis	In terms of health promotion, #dermatologist’ has 867.6 million views, #acne - 4.5 billion views, #eczema - 51.3 million views, #sunscreen -282.5 million views, #spf - 96.1 views, #skincancer - 38.2 million views and #tanningbed -18.9 million views.	Dermatology1	Low
Southwick et al. (2021)	USA	To characterise COVID-19 content posted by users and disseminated via TikTok.	Covid-19 videos	Content Analysis	One in four videos tagged with #coronavirus featured health-related content.	Covid-19	Acceptable
					Video Characteristics: Most videos contained "humour/parody," but 15% evoked "fear" and 6% evoked "empathy". The proportion of shares and comments for "misleading and incorrect information" featured in videos was lower in March than in January and February 2020. There was no statistical difference between the share and comment counts of videos coded as "incorrect/misleading" and "(factually) correct" over the entire time period.		
Unni & Weinstein (2021)	USA	To conduct a systematic analysis of trending TikToks related to COVID-19 over a 2-month period.	Covid-19 videos	Thematic Analysis.	In both month 1 and 2, trending COVID-19 related TikToks mostly frequently contained everyday circumstances and/or relatable commentary on Pandemic Life. Health-Promoting TikToks were more common than those demonstrating Risky or Concerning Health Practices (58:13) and were more common in Month 1. Advertisements and sponsored campaigns also shaped trending COVID-relevant content.	Covid	Acceptable
Villa-Ruiz et al. (2021)	USA	To characterize the content, sources, and reliability of the most popular dermatology videos on TikTok.	Dermatology videos	content analysis	Content creators were mostly patients (48.0%), board-certified dermatologists (25.8%), and estheticians (4.8%). Dermatologists were the main source of educational videos (41.3%), adhering to AAD guidelines 96.8% of the time.	Dermatology	Low
					The most common theme in videos was patient experiences/testimonies, followed by education, clinical demonstrations/live procedures, and product reviews/nonpaid advertisements. One-third (33.3%) of educational videos were posted by patients. Educational videos posted by board-certified dermatologists had a 50.1% greater probability of being reliable.		
Xu et al. (2021)	USA	To review the nature and quality of TikTok videos about PCa using validated metrics.	Prostate cancer TikTok videos	content review	Most videos were not created by those in healthcare or personal experience with PCa. Most posts were focused on awareness-raising or paying tribute to individuals with PCa. Most posts did not contain any real health information for the public. Of those that did contain “educational “health content, about half contained significant misinformation.	cancer	Low
Zheng et al. (2021)	USA	Our objective was to assess the quality of acne-related medical information present on TikTok, the world's fastest growing social media platform.	Acne TikToks	content analysis	The mean content quality of videos was low. Nine videos produced by United States board-certified physicians did have a higher content quality than videos by non-physicians. However, major shortcomings exist in both doctor and non-doctor created TikToks, eg. failure to cite information sources, discuss treatment risks, and provide support for shared decision-making.	Dermatology	Low

### Study 2

Spanning a range of account types that are popular in Ireland – public health accounts (*n* = 2), charity accounts (*n* = 5) and personal creator accounts (*n* = 22) – the overall data analysis covered TikTok content that totalled the following key engagement metrics: likes *n* = 2,588,181; comments *n* = 13,775; and shares *n* = 21,254. The summary characteristics illustrate differences between official public health accounts, TikTok personal accounts (i.e., creators), and charity accounts. Across the dialogic loop measure, in addition to TikTok’s suite of audio visual features, TikTok creators were the most engaging and interactive – this also accords with their higher followers. A full breakdown of each of these characteristics across each video and account is provided in Table 3, broken down by account types.

**Table 3.** Content analysis output table.

Account & Hashtag	Likes, Comments, Views and Shares	Video characteristics (8)	Video summary	Video length	Dialogic loop: (is a question posed in the title, content of the video or a pinned video?) Y = 1; N = 0 Video reply; Reply to comments - Yes or No (no numbers) Sharing of links: number the amount links (n= __)
Category 1. Public Health Accounts					
1.@hselive https://vm.tiktok.com/ZMdvhdYew/	Likes: 27.5k Comments: disabled Views; 796.1k Shares: 350	Informative - ad Public Health Content: Covid How is message delivered? Ad format. Voiceover, video, text at the end Title on video? Yes, 2 relevant hashtags. Text print? No, but there is closed captioning of voiceover Graphics? No Content creator? No Sounds used? Promoted music			Is a question posed in the title, content of the video or a pinned video? N/A Video reply; Reply to comments - N/A Sharing of links: number the amount links - N/A
2. @hselive https://vm.tiktok.com/ZMdvh1x2r/	Likes: 23.8k Comments:disabled Views: 743.1k Shares: 322	Informative - ad Public health content: covid How is message delivered? Ad format. Voiceover, video, text at the end Title on video? Yes, 2 relevant hashtags. Text print? No Graphics? yes Content creator? No Sounds used? Promoted music			Is a question posed in the title, content of the video or a pinned video? N/A Video reply; Reply to comments - N/A Sharing of links: number the amount links - N/A
Category 2. Charities					
1. Spunout https://vm.tiktok.com/ZMdvkFJFP/	Likes: 1990 Comments:42 Views: 61.7k Shares: 32	Public health content: Lgbt support, Mental health information How is message delivered? Person talking to the camera. Plain background. Text boxes. Closed captioning. Title on video? Yes, 1 relevant hashtag. Text print? Y Graphics? yes Content creator? No Sound: original sound			Creator likes comments? Yes Is a question posed in the title, content of the video or a pinned video? 1 (Yes) Video reply; Reply to comments - Yes Sharing of links: number the amount links - N0
2. Irish cancer society https://vm.tiktok.com/ZMdvkurd5/	Likes: 25 Comments: 1 Views: 690 Shares: 1	Ad informative Public health content: Cancer - Information on what is out there How is message delivered? Voiceover. Video. Closed captioning. Title on video? yes, with 3 cancer hashtags Text print? No Graphics? No Content creator? No Sound: Original sound			Creator likes comments? No Is a question posed in the title, content of the video or a pinned video? No Video reply; Reply to comments - No Sharing of links: number the amount links - No
3. @Jigsaw_ymh https://vm.tiktok.com/ZMdvBJnWr/	Likes: 79 Comments:0 Views:993 Shares: 0	not an ad. Public health content: Mental health, self love. How is message delivered? Text boxes.. Appear in time to song, dance Title on video? just 5 hashtags. Text print? Y Graphics? No Content creator? Yes (not a collab, the girl just knows how to engage with the format) dance/engagement with sound: Yes sound: Sarahplus3x - queen of random (popupar sound)			Creator likes comments? N/A Is a question posed in the title, content of the video or a pinned video? N/A Video reply; Reply to comments - N/A Sharing of links: number the amount links - N/A
4.@belongtoyouthservices https://vm.tiktok.com/ZMdvB9Kq3/	Likes: 5376 Comments: 0 Views:222.5k Shares: 1	Mental health ad Public health content: Mental health LGBT youth group How is message delivered? Graphic video promoting self-care with text. Promotion message about joining the lgbt- youth group in the title. Title on video? Yes. No relevant hashtags Text print? N Graphics? Yes Content creator?No dance/engagement with sound: No Original sound			Creator likes comments? N’/A Is a question posed in the title, content of the video or a pinned video? N/A Video reply; Reply to comments - N/A Sharing of links: number the amount links - N/A
5.Turn2me https://vm.tiktok.com/ZMdvSWpMJ/	Likes: 35 Comments: 2 Views: 573 Shares:6	Promotion of turn2me Mental health ad Public health content: Mental health, help-seeking How is message delivered? Person pointing to text bubbles. Title on video? Yes. 1 relevant hashtag Text print? Y Graphics? N Content creator? Y dance/engagement with sound: N Original sound			Creator likes comments? Y Is a question posed in the title, content of the video or a pinned video? No Video reply; Reply to comments - No Sharing of links: number the amount links - No

3. Personal tiktokers					
1. #irish #carcrash @rachelhughes198 https://vm.tiktok.com/ZMRxHP5Hj/	Likes:796 likes, Comments: 14 Views: 11.4k Shares:24	Funny video about damaged car Public health content: Humorous take on consequences of car crash Info conveyed? N. Title on video: N. (14 hashtags) Text print? Y Graphics? Y Content creator in video? N dance/engagement with sound: N Lighting effect: N Creator acting: Y Sound: Original	Image of damaged white car appears in video. In text print on screen, "To the bastard that reversed into my car in a shopping centre car park and drove off (clown face emoji)." Voiceover reads out the text print. Image cuts to the classic image of Liam Neeson in the film "Taken" saying "I will find you, and I will kill you."	N/A	Creator likes comments? Yes Creator comments? Yes Is a question posed in the title, content of the video or a pinned video? No Video reply: Reply to comments - No, Yes Sharing of links: number the amount links - No
2. #irish #murder @pjmccarthy https://vm.tiktok.com/ZMRxHp6ER/	Likes:30.2k Comments: 1248 Views: Info Unavailable Shares:916	Funny video about meeting a man convicted of murder Public health content: Humorous take on murder Info conveyed? N. Title on video: Y, (Casual chats up in this bi$h”.(6 hashtags) Text print? Y Graphics? Y Content creator in video? Y dance/engagement with sound: N Lighting effect: N Acting: N Sound: “What the fuck was that” - champagnemami. 104.8k videos	Video of content creator with text print on screen that states “casually touring West Cork”. Video cuts to images of creator and a renownedmurder suspect. Text print states “starts a chat with convicted murderer Ian Bailey”	N/A	Creator likes comments?Yes Creator comments? Yes Is a question posed in the title, content of the video or a pinned video? Yes, pinned comment Video reply; Reply to comments - No, Yes Sharing of links: number the amount links - No
3. #irish #firstaid @classycody https://vm.tiktok.com/ZMdvAvDtt/	Likes: 237 Comments: 38 Views: 3874 Shares: 1	Funny video Public health content: First aid Info conveyed? No Title on video? Yes. "Everyone stay calm, I know first aid" (10 hashtag) Text print? N Graphics? N Content creator? Y dance/engagement with sound: N Lighting effect: N Acting: N Sound: Original sound	How is message delivered? Video of girl attempting cpr. She is laughing as she gives the CPR dummy a smack on the face.	N/A	Creator likes comments? Yes Creator comments? Yes Is a question posed in the title, content of the video or a pinned video? No Video reply; Reply to comments - No, Yes Sharing of links: number the amount links - No
4. # irish #smoking @lilgeep https://vm.tiktok.com/ZMdvA9URH/	Likes:5020 Comments: 26 Views: 45.2k Shares: 78	Funny video Public health content: Smoking peer pressure Smoking peer pressure expectations vs reality Info conveyed? Y. peer pressure is not what it looks like Title on video? Yes, “Expectation vs reality”. 5 relevant hashtags Text print? Y Graphics? N Content creator? Y dance/engagement with sound: N Lighting effect: Y Acting: y Sound: Meduza & Becky Hill & Goodboys - lose control 7.1m	Text print: Expectations. Content creator begins to role play two sides of a conversation. Character smoking: “You’re gonna smoke this fag”. Non-smoker: “Ah I’m alright though, thanks but I don't want one.” Smoker: “Listen right here pal, you don’t smoke this fag I’m gonna tell everyone in school you’re an absolute weirdo.” Non-smoker: *coughs* Text print: Reality: Smoker: “Oh sorry pal did you want one?” Non-smoker: “No, sorry, I don’t smoke”. Smoker: Fair play, fair play you’re a better man than myself. I vcouldn’t even give up these yokes for a day like. You’re much better than I could ever be, fair play”	32 seconds	Creator likes comments? Yes Creator comments? Yes Is a question posed in the title, content of the video or a pinned video? No Video reply; Reply to comments - No, Yes Sharing of links: number the amount links - No
5. #irish #alcohol ‘Shanejkennedy https://vm.tiktok.com/ZMdvDRtFg/	Likes:199.2k Comments: 1322 Views: 1.2M Shares: 7587	Information: strongest alcoholic drinks in the world Public health content: Alcohol promotion Info conveyed? Y. Title on video? Yes “Strongest spirits part 2”. 14 relevant hashtag Text print? Y Graphics? Y Content creator? Y dance/engagement with sound: N Lighting effect: N Acting: N Sound: Original sound. (not really a sound, its voiceover)	How is message delivered? Video of guy explaining the alcohol content of the strongest spirits in the world. , Pictures of alcohol inserted and corresponding text boxes convey the alcohol percentage of each spirit. ****************	48 secs	Creator likes comments? Yes Creator comments? Yes Is a question posed in the title, content of the video or a pinned video? No Video reply; Reply to comments - No, Yes Sharing of links: number the amount links - No
6. #irish #mentalillness @saoishell https://vm.tiktok.com/ZMdvPDbJ6/	Likes:150.3k Comments: 2309 Views: 696.5k Shares:2261	Depression humour Public health content: Shares info about depression - being too depressed to brush teeth, effect on teeth. Info conveyed? Y. Title on video? Just 8 relevant hashtag Text print? Y Graphics? N Content creator? Y dance/engagement with sound: Y Lighting effect: N Acting: N Sound: Be nice to me -the front bottoms (humorous effect when combined with video) 48.4k	How is message delivered? Video of girl lip syncing to song. Text print appears “Me, too depressed to perform basic self care most of the time from 12 onwards”. Video cuts to new scene, girl gives peace sign (gesture) and text box appears with the words “the enamel on my teeth”. Music used for humorous effect. *************	N/A	Creator likes comments? Yes Creator comments? Yes Is a question posed in the title, content of the video or a pinned video? No Video reply; Reply to comments - No Sharing of links: number the amount links - No
7. #irish #mentalhealth @benzoooz https://vm.tiktok.com/ZMRxHusJM/	Likes: 574.3k Comments: 3838 Views: 2.3m Shares:1349	Mental health humour Public health content: Shares info about being in a mental health hospital Info conveyed? Y. Title on video? Just 8 relevant hashtag Text print? Y Graphics? N Content creator? Y dance/engagement with sound: Y Lighting effect: N Acting: N Sound: Original sound -seanioworld	Video of girl giving peace sign (gesture), text boxes to tell story about being in a mental health hospital, Humorous music playing.	N/A	Creator likes comments? Yes Creator comments? Yes Is a question posed in the title, content of the video or a pinned video? No Video reply; Reply to comments - No Sharing of links: number the amount links - No
8. #irish #depressed @arachnerd_cosplay https://vm.tiktok.com/ZMR8UmRXY/	Likes: 930 Comments: 17 Views: 3421 Shares:11	Mental health humour Public health content: Raises awareness of the experience of coming out of a depressive episode Info conveyed? Y. Title on video? Just 5 relevant hashtags Text print? Y Graphics? N Content creator? Y dance/engagement with sound: Y Lighting effect: N Acting: N Sound: Sound: star.la 2354 videos “I guess you wonder where I’ve been”	“Ghosting everyone during a depressive episode and coming back like nothing happened” is the text print on screen to a blank background. Guy pops up on screen “guess you wonder where I’ve been” sound plays.	N/A	Creator likes comments? Yes Creator comments? No Is a question posed in the title, content of the video or a pinned video? No Video reply; Reply to comments - No Sharing of links: number the amount links - No
9. #irish #anxiety @wonder_art https://vm.tiktok.com/ZMRxHwaGC/	Likes:17.9k Comments: 975 Views: 69.9k Shares:227	Mental health humour Public health content: Connects to other people experiencing difficulties getting mental health help in Ireland. Info conveyed? N. Title on video? Y “best of luck to us all, I hope someone gets actual help” Text print? Y Graphics? N Content creator? Y dance/engagement with sound: Y Lighting effect: N Acting: N Sound: “Mr. Black is a very good singer, 79.6k videos.	Video of girl dancing to “Na, na, na, na,na, na, na. “We’re completely fucked, we’re completely fucked“. Text on screen says “everyone trying to get mental health help in Ireland”	N/A	Creator likes comments? Yes Creator comments? Yes Is a question posed in the title, content of the video or a pinned video? No Video reply; Reply to comments - No Sharing of links: number the amount links - No
10. #irish #edawareness @eimearmc9 https://vm.tiktok.com/ZMR8Ugrmw/	Likes:5607 Comments: 109 Views: 43.6k Shares:0	Eating disorder humour Public health content: There are only 2 foods she doesn’t feel guilty eating Info conveyed? Y. Title on video? “I do not promote disordered eating(laughing face)” Text print? Y Graphics? N Content creator? Y dance/engagement with sound: Y Lighting effect: N Acting: N Sound : Gemmacollinsdiva, 49.5k views	Sound: gemmacollinsdiva, 51.6k videos How is message delivered? Video of girl with text popping up on screen: “Foods I feel no guilt after eating.” then the following text boxes appear: “1-grapes”, “2 yoghurt” Sound playing is “I’ve got not one, not two, oh no it is two” - Gemma Collins sound	N/A	Creator likes comments? Yes Creator comments? Yes Is a question posed in the title, content of the video or a pinned video? No Video reply; Reply to comments - No Sharing of links: number the amount links - No
11. #irish #bipolar @cymbaltaslut https://vm.tiktok.com/ZMR8UEPSh/	Likes:204 Comments: 2 Views: 708 Shares:2	Bipolar stigma humour Public health content: mental illness Info conveyed? Y. Title on video: “get both things out of the way at once, amirite”, 7 hashtags relevant Text print? Y Graphics? N Content creator? Y dance/engagement with sound: Y Lighting effect: N Acting: N Sound: i love you 22.4k videos	Text print appears on screen: “Introducing myself to people as a lesbian with bipolar disorder” Content creator is mouthing the “Guys I need to tell you something, I think I’m homosexual but I’m also addicted to cocaine” awkward voice sound.	N/A	Creator likes comments? Yes Creator comments? No Is a question posed in the title, content of the video or a pinned video? No Video reply; Reply to comments - No Sharing of links: number the amount links - No
12. #irish #bodyimage @oxrobyn.sucksxo https://vm.tiktok.com/ZMR8UKAvP/	Likes: 1193 Comments: 36 Views: 7739 Shares:16	Fun body positivity celebration Public health content: Info conveyed? N. Title on video? Y (still a baddie tho) 9 relevant hashtags Text print? Y Graphics? N Content creator? Y dance/engagement with sound: Y Lighting effect: N Acting: N	Sound: folding chair 37.2k videos “I got a perfect body” song plays while content creator dances to the music in bikini, celebrating their body.	N/A	Creator likes comments? Yes Creator comments? No Is a question posed in the title, content of the video or a pinned video? No Video reply; Reply to comments - No Sharing of links: number the amount links - No
13. #irish #sexual health @accidentalainbow https://vm.tiktok.com/ZMR8f2Gt9/	Likes:4976 Comments: 77 Views: 49.7k Shares:137	Sexual health testing information Public health content: Info conveyed? Y. Title on video? Y (seggsual health ) 8 relevant hashtags Text print? Y Graphics? N Content creator? Y dance/engagement with sound: Y Lighting effect: N Acting: N Sound: Sexual (NEIKED) 348 videos. Also a popular song	Demonstrates the process of getting a sexual health test. Text pops up detailing each stage. Creator demonstrates each stage, going through the process himself. Humorous	Video length - 35secs	Creator likes comments? Yes Creator comments? Yes Is a question posed in the title, content of the video or a pinned video? No Video reply; Reply to comments - No Sharing of links: number the amount links - No
14. #irish #vaccine @killersundy https://vm.tiktok.com/ZMR8f8RGF/	Likes:58.1k Comments: 258 Views: 292.1k Shares:2570	Mocking the Irish government vaccine rollout Public health content: vaccine humour Info conveyed? N. Title on video? Y (“Irish government be ordering vaccines like it’s takeaway”) Text print? Just closed captions Graphics? N Content creator? Y dance/engagement with sound: Y Lighting effect: N Acting:Y Sound: Original Sound	The actor role plays how the government debates amongst itself when trying to order vaccines for the country. Has a pretend phone conversation. Humorous	Video length - 59 secs	Creator likes comments? Yes Creator comments? Yes Is a question posed in the title, content of the video or a pinned video? No Video reply; Reply to comments - No Sharing of links: number the amount links - No
15. #irish ∼dentist https://vm.tiktok.com/ZMR8fey37/	Likes:3654 Comments: 95 Views:125.8k Shares:80	Public health content: Shows how to remove tonsil stones. Humorous Info conveyed? Y.(from an actual doctor) Title on video? N (10 hashtags) Text print? Y Graphics? N Content creator? N dance/engagement with sound: Y Lighting effect: N Acting: N Sound: WAP - cardi b(feat Megan Thee Stallion) 75.6k videos.	Shows the back of someone’s mouth with a tonsil stone. Gives the correct anatomical labels. Shows how to remove with cotton bud. Music plays for entire video. Health communication is through video demonstration and text boxes.	Video length - 59 secs	Creator likes comments? No Creator comments? Yes Is a question posed in the title, content of the video or a pinned video? No Video reply; Reply to comments - No Sharing of links: number the amount links - No
16. #irish #asthma @raychill-98 https://vm.tiktok.com/ZMRx9RqcP/	Likes:4411 Comments: 43 Views: 36.2k Shares:66	Public health content: humorous asthma info from a doctor Info conveyed? Y.(from an actual doctor) Title on video? N (13 hashtags) Text print? Y (pot calling kettle black denise x) Graphics? N Content creator? Y dance/engagement with sound: N Lighting effect: N Acting: Y Sound: none	Humorous. Retells a story where she went to take a puff of her inhaler but her mam called her dramatic and said that everyone will think she has covid.	55 secs	Creator likes comments? Yes Creator comments? No Is a question posed in the title, content of the video or a pinned video? No Video reply; Reply to comments - No Sharing of links: number the amount links - No
17. #irish #allergy @irishmammylife https://vm.tiktok.com/ZMR8fbxuH	Likes:5839 Comments: 309 Views: 56.7k Shares:111	Allergy humour Public health content: Humorous take on hay fever symptoms. Info conveyed? Y. Title on video? N (13 hashtags) Text print? Y Graphics? N Content creator? Y dance/engagement with sound: Y Lighting effect: N Acting: Y Sound: maria.pintoo 219.4k videos	Acts out to “I’m going insane, yeah” music. Does dramatic expressions. Text boxes pop up eg. “When you can’t enjoy your summer with hay fever. “Then different symptoms pop up in text boxes which she acts out eg. “itchy puffy burning watery eyes”	N/A	Creator likes comments? Yes Creator comments? Yes Is a question posed in the title, content of the video or a pinned video? No Video reply; Reply to comments - No Sharing of links: number the amount links - No
18.#irish #skin @.sophiekeegan https://vm.tiktok.com/ZMR8fxnuH/	Likes: 94.4k Comments: 433 Views: 660.5k Shares: 1702	Skincare humour Public health content: Info conveyed? N. Title on video? Y” the amount of time a boy has asked me what on ur face is astronomical” (3 hashtags) Text print? Y Graphics? N Content creator? Y dance/engagement with sound: Y Lighting effect: N Acting: N Sound:_.chenqing._73.6k videos	“The irish secret to clear skin” pops on screen, with creator acting to music underneath. Then shows lots of images of girls with sudocreme on their face. In time to music.	N/A	Creator likes comments? Yes Creator comments? Yes Is a question posed in the title, content of the video or a pinned video? No Video reply; Reply to comments - No Sharing of links: number the amount links - No
19. #irish #cancer @bernadettehagans https://vm.tiktok.com/ZMR8fCf2G/	Likes: 111.3k Comments: 1062 Views: 1.7m Shares: 2333	Cancer humour Public health content: Info conveyed? N. Title on video? ”feel like Alex” (10 hashtags) Text print? Y Graphics? N Content creator? Y dance/engagement with sound: Y Lighting effect: N Acting: Y Sound: interior crocodile alligator 527.6k videos	“Beating cancer” humour. Text print: “When cancer tried to get rid of me but I won in the Gulag” Creator walks onto the screen with dark sunglasses on	N/A	Creator likes comments? Yes Creator comments? Yes Is a question posed in the title, content of the video or a pinned video? No Video reply; Reply to comments - No Sharing of links: number the amount links - No
20. #irish #diabetes @hi_im_lee https://vm.tiktok.com/ZMR8fq8yG/	Likes: 1534 Comments: 12 Views: 7575 Shares: 5	Public health content: Diabetes humour Info conveyed? N. Title on video? Y - Get with me (9 hashtags) Text print? Y Graphics? N Content creator? Y dance/engagement with sound: Y Lighting effect: N Acting: Y Sound: juanss152, 57 videos	Junk food on table. “If you like guys with type 2 diabetes” appears on screen. Then creator appears, flirting with the camera	N/A	Creator likes comments? No Creator comments? No Is a question posed in the title, content of the video or a pinned video? No Video reply; Reply to comments - No Sharing of links: number the amount links - No
21. #irish #nutrition @@michneilly https://vm.tiktok.com/ZMR8f93cE/	Likes: 189.0k Comments: 572 Views: 940.3k Shares: 1108	Public health content: Nutrition Advice Info conveyed? Y. Title on video? ”CALORIE DEFICIT MADE SIMPLE” (7 hashtags) Text print? Y Graphics? N Content creator? Y dance/engagement with sound: Y Lighting effect: N Acting: N Sound: SHaxicula (toxic x Love Shack x Dragula) 696.3k videos	3 tips to make a calorie deficit easier to stick to. Talks to camera, text print also pops up.	Video length: 49 secs	Creator likes comments? Yes Creator comments? Yes Is a question posed in the title, content of the video or a pinned video? No Video reply; Reply to comments - No Sharing of links: number the amount links - No
22 .#irish #health @tadghoody https://vm.tiktok.com/ZMR8fhfPF/	Likes: 1129.1k Comments: 980 Views: 711.0k Shares: 670	Public health content: The fitness grind Info conveyed? N. Title on video? ”Day 70” (11 hashtags) Text print? Y Graphics? N Content creator? Y dance/engagement with sound: Y Lighting effect: N Acting: N Sound: sweater weather x another love DJ Lilli 151.9k videos	Promotes taking care of physical health. Text box shows “DAY 70/79”video footage of him training and working out.	N/A	Creator likes comments? Yes Creator comments? Yes Is a question posed in the title, content of the video or a pinned video? No Video reply; Reply to comments - No Sharing of links: number the amount links - No
Results:	Total likes = 2,588,181 Total comments = 13775 total shares = 21,254	Summary characteristics. -Public health: 0/2 use humour 2/2 conveyed health info 0/2 used text print 0/2 the content creator appeared 0/2 engaged with the sound used either through dance or the timing of the words in the song 0/2 content creators acted in the TikToks 0/2 used a popular sound on the platform -Charities: 1/5 use humour 4/5 conveyed health info 3/5 used text print 2/5 the content creator appeared 1/5 engaged with the sound used either through dance or the timing of the words in the song content creators acted in the TikToks 0/5 used a popular sound on the platform 3/5 videos used a sound popular on the platform. -Personal Tiktoks: 14/22 conveyed health info 20/22 used text print 20/22 the content creator appeared 16/22 engaged with the sound used either through dance or the timing of the words in the song 7/22 content creators acted in the TikToks 17/22 used a popular sound on the platform 16/22 used humour 18/22 videos used a sound popular on the platform. The mean likes of videos using a sound popular on the platform: 132,419 likes. The mean likes of videos not using a sound popular on the platform: 51,161 likes.		Mean = 48.14 seconds	20/22 creators liked comments 17/22 creators replied to comments 2/5 charities liked comments 1/5 charities responded to comments The HSE did not enable comments Dialogic loop: No dialogic loop was used by charities / the HSE. (i.e., is a question posed in the title, content of the video or a pinned video?) Y = 1; N = 0 37% Yes Video reply; Reply to comments - Yes or No (no numbers) 68% Yes Sharing of links: number the amount links (n= 0 )

## Overall discussion

This two-part study aimed to provide what we think is the first systematic review of TikTok and its use within mental and public health for young people, in addition to contextualising this with a content analysis. Study 1 collated 24 studies across a range youth mental and public health issues, demonstrating the versatility of TikTok in the current ecosystem. Study 2 provided a follow-up example of how TikTok is being used within an Irish context, and found high engagement rates across public health, charity and personal creator TikTok accounts (*n* = 29); but with personal creator accounts significantly utilising the full range of features of the platform more so than other account-types. Taken together, and in light of the young user-base of TikTok, these results tentatively indicate the creative and novel opportunities for leveraging the platform for positive mental and public health outcomes. Nonetheless, given the ubiquitous nature of TikTok amidst the scant evidence for its effects on CYP, caution must still be reserved for its potentially negative outcomes. Indeed, many of the included studies in the review found that TikTok was being used by both professionals and creators alike to communicate short-form audio-visual content relating to key issues for young people, including acne, pandemic information, and eating disorders. However, professionally accredited information was often in the minority. This presents a dilemma to the wider TikTok audience – to what extent can a pre-teen userbase distinguish between reputable mental and public health professional information versus non-professional equivalents? Our follow-up content analysis using Irish hashtags elicited a highly diverse sample of accounts that addressed a range of mental and public health issues. TikTok is clearly seen as an outlet to interact about many serious issues, but often using humour as the means of delivery. Personal TikTok creators significantly used the full range of features to execute their message, whereas charity and public health accounts only engaged a limited amount of features. Consequently, their engagement metrics were demonstrably lower. For example, dialogic loop was consistently higher across all personal TikTok accounts thereby encouraging the respective audience to maintain engagement. Creative integration of information, titles, text, graphics, dance, sound syncing, lighting, and acting allowed personal creators to elevate their message; whereas other accounts typically relied on more traditional social media formats. This underscores a key difference between uptake and novelty of effective TikTok use – until mental and public health stakeholders critically understand the vast functionalities of TikTok, they will likely continue to miss the opportunity to use it as an efficient means of communicating with CYP.

## Limitations

Compared to the broader literature concerning other dominant social media platforms, this study found relatively fewer papers from which to offer generalisable points. Additionally, the highly variable quality of studies in the systematic review and the disproportionate reliance on certain methodologies from predominately North American researchers should also be critically considered when interpreting our overall findings. With respect to Study 2, there are clear drawbacks from conducting an analysis on a limited set of hashtags blended with our search terminology. This method of discovering TikTok content may not be reflective of the complex ways in which CYP encounter mental and public health information on the platform. Larger automated means of distilling a wider corpus of data related to such topics would likely achieve more representative results.

## Future research

Unquestionably, future research should seek to apply a wider range of methodological approaches to understanding the mental and public effects of TikTok on CYP. Controlled studies examining relationships between behavioural change and TikTok engagement across different topics will advance our understanding of the precise factors influencing either positive or negative outcomes. More multidisciplinary in-depth studies that blend data science approaches to collecting qualitative data, alongside clinical interpretation, will assist in building best practice guidance for professionals who would like to use TikTok to convey important and likely impactful information to CYP.

## Recommendations for mental health professionals

Due to the absence of any established evidence base for mental health professionals interested in using TikTok, the following ethically-minded best practice recommendations are provided based on the available literature and current observations of how TikTok functions:1) Establish a clear a prior content policy as to the purpose of engaging TikTok for communication purposes – why, and for whom, is the proposed content appropriate? A clear disclaimer about content being for educational and not directive purposes should be communicated. All content should be aligned with the respective professionals’ governing body, and any relevant social media policy therein. 2) To optimise the engagement, professionals should ensure that the full range of TikTok features are utilised such that content is not merely a replication of multimedia used on other platforms. 3) Ongoing critical analysis should be applied to engagement data – are the analytics reflective of in-depth user engagement that may lead to positive outcomes, such as increased help-seeking behaviours, higher uptake of services, or application of material to real-world scenarios. On the other hand, what are the consequences of providing TikTok content given the difficulty of knowing how the content is specifically used?
